# Surgery of the Peritoneum

**Published:** 1904-06-25

**Authors:** 


### SURGERY OF THE PERITONEUM.

**Chronic Adhesive Sclerosing Peritonitis**, according to Wetherill,[1] presents the following points:—(1) The disease is rare. (2) The inability to unite the shrinking peritoneum when once divided during the course of an operation is pathognomonic. (3) The disease is essentially chronic. (4) It is of great importance to cover all denuded surfaces when, before or during the operation, chronic adhesive sclerosing peritonitis is diagnosed. (5) The characteristic subperitoneal connective tissue hyperplasia is the pathological feature of the disease. (6) The shrinking or contraction of the peritoneum caused by it is the clinical feature of the disease. (7) The possible significance of a somewhat similar pathological process taking place in the skin, coincident with unusual gastro-intestinal symptoms is otherwise unaccounted for. (8) There are various sequelæ of a serious character incident to its surgical treatment. (9) There is a possibility of accomplishing much through medicinal measures, when the etiology of the disease is better understood. Potassium iodide and inunctions of mercury are worthy of careful consideration and trial in any suspected case of plastic peritoneal sclerosis.

**Tuberculous Peritonitis.**—Lloyd[2] does not think it worth while to operate upon any patient who has marked pulmonary tuberculosis or who gives evidence of a general tuberculosis. On the other hand, in cases of acute miliary tuberculosis he thinks great benefit might accrue from an abdominal incision and washing out of the whole abdominal cavity with a full-strength solution of hydrogen peroxide. It is remarkable how each tuberculous nodule will stand out prominently after this application. In these cases of miliary tuberculosis he believes the operation should be done as soon as the diagnosis can be made. By operation, the fluid is evacuated and an opportunity afforded for washing out the abdominal cavity with the solution of hydrogen peroxide. If the wound is left open, as he prefers in all cases, it prevents a second distension of the abdomen, and favours a marked improvement in the general health. In the case of chronic caseous or ulcerating tuberculosis the performance of the operation depends largely upon the seat of the disease and its extent. If only a small number of isolated intestinal glands are involved it might be possible markedly to benefit, or even to cure, the patient. In the case of chronic fibroid tuberculosis he does not believe surgical interference is advisable. He considers that operation for tuberculous peritonitis affords the patient an opportunity to gain in strength and nutrition which would not be possible under any medical treatment.

**Closure of the Abdomen after Laparotomy.**—Premising that the tier suture is generally regarded as the nearest approach which we can attain to the natural conditions existing before operation, and that this with some combination of the mass (or through and through) suture, probably offers the strongest barrier possible to hernia and to infection through accumulation of fluids within the walls, Higgins[3] points out that the tier suture is chiefly objected to because of the danger of the so-called dead spaces; and that the mass or retention sutures passed through the whole of the layers, including the skin, not infrequently irritate and cut through the latter, giving rise to additional scars on the abdomen. On the other hand, the early removal of these through and through sutures is apt to end in some separation of the edges of the wound entailing delayed union and a wider scar. With a view, then, to the minimising of the usual dead space between the layers, and the prevention of the traction scarring of the skin, Higgins has introduced the use of a narrow metal plate with straight parallel edges. This plate is a strip of thin nickel-plated copper, about one-thirtieth of an inch thick. It is stiff enough not to be bent by the sutures, but it is easily bent by the fingers in case it needs to be adjusted to any inequalities in the surface of the abdomen. In practice it was found inexpedient to employ either perforations or serrations in the metal plate, chiefly on account of the extra time taken up in putting in the sutures with such care as to bring them opposite the perforations or serrations. The plain strip of metal above described is found quite satisfactory, the sutures hold equally well, and the plate does not slip if properly fixed. The mass sutures of silkworm gut are introduced by double-threaded large curved needles. The peritoneum is then closed by a running suture of catgut, the fascia by another gut suture, and the skin by a subcuticular suture of fine silkworm gut. The mass sutures are finally tied over the metal guard, which is separated from the abdominal wall by a pad of folded sterile gauze. For this method it is claimed: (1) It in no way interferes with the perfect and aseptic healing of the wound. (2) It effectually controls all oozing of blood and serum into the wound, so that it is rarely necessary to ligate any vessels; the accumulation of all collections of blood within the layers of the abdominal wall is thereby prevented. (3) The pressure of the metal guard promotes adhesion between the layers in the abdominal wall.

[1] Med. Rec. New York, Jan. 23, 1904, p. 157. [2] Ibid., Feb. 13, 1904, p. 274. [3] Glas. Med. Journ., Feb. 1904, p. 163.

